# Developmental dynamics of mitochondrial fission and fusion proteins in functionally divergent skeletal muscles of goat

**DOI:** 10.14814/phy2.16002

**Published:** 2024-06-03

**Authors:** Sunil Pani, Unmod Senapati, Benudhara Pati, Bijayashree Sahu, Gourabamani Swalsingh, Punyadhara Pani, Subhasmita Rout, K. Gopinath Achary, Naresh C. Bal

**Affiliations:** ^1^ School of Biotechnology KIIT University Bhubaneswar Odisha India; ^2^ Imgenex India Pvt. Ltd. Bhubaneswar Odisha India

**Keywords:** mitochondria, oxidative capacity, protein–protein interaction, proximity ligation assay, skeletal muscle

## Abstract

During skeletal muscle development, the intricate mitochondrial network formation relies on continuous fission and fusion. This process in larger mammals differs from rodents, the most used animal models. However, the expression pattern of proteins regulating mitochondrial dynamics in developing skeletal muscle remains unexplored in larger mammals. Therefore, we characterized the cellular expression and tissue‐level distribution of these proteins during development taking goat as a model. We have performed histological and immunohistochemical analyses to study metabolic features in various muscles. Neonatal muscles display uniform distribution of mitochondrial activity. In contrast, adult muscles exhibit clear distinctions based on their function, whether dedicated for posture maintenance or facilitating locomotion. Mitochondrial fission proteins like DRP‐1, MFF, and fusion proteins like MFN‐1 and 2 are abundantly expressed in neonatal muscles. Fission proteins exhibit drastic downregulation with limited peripheral expression, whereas fusion proteins continue to express in a fiber‐specific manner during adulthood. Locomotory muscles exhibit different fibers based on mitochondrial activity and peripheralization with high SDH activity. The proximity ligation assay between MFN1 and MFN2 demonstrates that their interaction is restricted to subsarcolemmal mitochondria in adult fibers while distributed evenly in neonatal fibers. These differences between postural and locomotory muscles suggest their physiological and metabolic properties are different.

## Graphical abstract

During the postnatal development of skeletal muscles, fission proteins exhibit drastic downregulation, while it is not significant for fusion proteins. While mitofusins display individual fiber‐specific localization, fission proteins are evenly distributed throughout the fibers. MFN1–MFN2 interaction is distributed uniformly in the neonatal fibers whereas predominantly localized to peripheral zones of adult fibers.

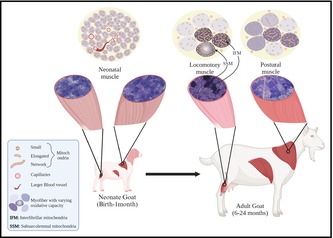

## 1 INTRODUCTION

The developmental regulation of the skeletal muscle (SkM) from uninucleated mesenchymal progenitor cells to multinucleated myocytes is a fascinating biological phenomenon. This developmental pattern exhibits unique variability from species to species depending on several factors like precociality, adulthood metabolism, and neonate‐to‐adult body mass ratio. To achieve this, their SkM undergoes profound remodeling as per their location in the body and postnatal function. Concurrently, their fibers acquire several unique structural‐functional properties including mitochondrial abundance and distribution, as well as cellular vascularization (Larsen et al., 2012; Pastoris et al., 2000; Pette, 2002). Our previous research using rat as a model has shown appreciable differences in fiber‐specific mitochondrial distribution between neonate and adult SkM (Senapati et al., 2023). However, SkM is a significantly heterogeneous organ and different muscle groups vary among each other based on their contractile and metabolic properties. SkM has been categorized as postural and locomotory exhibiting distinct protein expression pattern, which is even more pronounced in case of larger mammals. While most of the fibers in postural muscles acquire high oxidativeness, locomotory muscles exhibit a mixed population of oxidative as well as glycolytic fibers (Pette, 2002). The oxidativeness of the fibers is determined by the mitochondrial density, and the fiber composition of different muscle groups varies in a species‐specific manner (Curry et al., 2012; Missina et al., 2022). The limb muscles of burst runners like cheetahs, deer, and mice are rich in glycolytic fibers, whereas those of long‐distance movers like elephants are rich in oxidative fibers (Curry et al., 2012; Goto et al., 2013; Hyatt et al., 2010). Mammals (like humans, goats, and cattle) that are intermediate possess limb muscles containing a mixture of both oxidative and glycolytic fibers (Missina et al., 2022). Further studying SkM development in goats will bridge several gaps in knowledge like its contribution to whole‐body energy expenditure, differential role muscles from different locations, and nonshivering thermogenesis mechanisms in SkM. These questions are not possible to answer using mice as an animal model as they display a sustained expression of brown fat and it is a major contributor to whole‐body energy expenditure. In contrast, goats show a transient expression of brown fat, like humans during the perinatal period which suggests in both animals SkM play more significant roles than locomotion (Pani et al., 2022).

During the perinatal development of SkM; all the crucial metabolic apparatus like mitochondria, contractile apparatus, and sarcoplasmic reticulum (SR) undergo major reorganization (Pani et al., 2022). Initially, mitochondria are found to be arranged in individual structures and run in parallel to the sarcomere axes. But, a few days postnatal mitochondria become a highly branched network that wraps around the myofibrils (Katti et al., 2022). The formation of mitochondrial network varies across muscles, and appreciable differences can be observed between locomotory and postural muscles in rodent species (Katti et al., 2022; Senapati et al., 2023). It is suggested that all muscle groups exhibit a similar distribution of mitochondria inside the fiber during initial stages of development. After a few days of postnatal development, fibers become distinct with respect to their oxidativeness (Crupi et al., 2018; Larsen et al., 2012). The differentiation and maturation of SkM corroborates with the rapid increase in intramuscular vascularization as it supplies necessary nutrients and oxygen to the myofibers. Moreover, there is a prevailing notion that vascularization, physical activity, and oxidative capacity of SkM remain intricately intertwined with each other (Laughlin & Roseguini, 2008; Palstra et al., 2014). The oxidative capacity of the SkM is regulated by several factors, out of which mitochondrial dynamics is a primary determinant (Glancy et al., 2015; Mishra et al., 2015; Twig et al., 2008). Mitochondrial dynamics is the process by which mitochondria undergo regulated fission and fusion to fulfill the metabolic requirement of the individual muscle fibers (Mishra et al., 2015). Although several studies have shown the developmental differences in mitochondrial properties among muscle groups, a detailed study across full developmental window from both pre‐ and postnatal period is missing.

Mitochondrial fission is regulated by proteins like mitochondrial fission factor (MFF), dynamin‐related protein‐1 (DRP‐1), and fission 1 protein (FIS1), whereas mitochondrial fusion is mediated by proteins like mitofusin‐1 (MFN‐1), mitofusin‐2 (MFN‐2), and dynamin‐related GTPase/Optic Atrophy‐1 (OPA‐1). During perinatal development, all the organisms exhibit tremendous rates of fission and fusion cycles to increase the number and network of mitochondria (Ishihara et al., 2015; Kim et al., 2019; Touvier et al., 2015). While the fission‐associated proteins significantly enhance the number of mitochondria, the fusion‐associated protein forms the network of mitochondria around myofibril (Kim et al., 2019). Mitofusins are also suggested to establish the communication of mitochondria with the SR which is also a major factor in uplifting mitochondrial metabolism (Kim et al., 2019; Romanello, 2021). Most of the mitochondrial dynamics associated factors are already expressed before birth in larger mammals (Yan et al., 2013). Inspired by our previous study on rodent, we have expanded our current study into prenatal stages, including characterization of mitochondrial fission proteins. Additionally, we have taken up functionally (locomotory and postural) divergent SkM using goat as a model. Therefore, this study is first of its kind to address the SkM metabolic development in an elaborate manner from both prenatal and postnatal time periods in goat, which is physio‐metabolically closer to humans. This encompasses SkM vasculature, mitochondrial density, and proteins associated with mitochondrial dynamics. The findings of this study establish the time points of induction of vascularization, distinct oxidative phenotype of myofibers, and expressional distribution of mitochondrial dynamics proteins.

## 2 MATERIALS AND METHODS

### 2.1 Animal tissue collection, preservation, and sectioning

SkM tissue samples from different locations were obtained from animals of desired age (i. 45–55 days of prenatal age: Early gestation (EG); ii. 75–90 days of prenatal age: Mid gestation (MG); iii. 120–130 days of prenatal age: Late gestation (LG); iv. 1 week; v. 2 weeks; vi; 1 month; vii. Adult) at a goat farm (Manikstu Agro, Salebhata, Odisha, India) (Detailed in Table 1). The SkM were shortlisted on the basis of their contribution toward locomotion and posture maintenance, such as gastrocnemius (primarily locomotory), latissimus dorsi (function for both locomotion and posture maintenance), and trapezius (primarily postural). All the animal experimentation procedures were performed at Manikstu Agro, in accordance with the guidelines of the institutional animal ethics committee (IAEC) of the School of Biotechnology, KIIT with approval number: KSBT/IAEC/2019/MEET‐1/A13. The tissues were obtained from three female animals per age group for all the postnatal groups as the males are castrated below 1 month of age. For prenatal animals, samples were collected irrespective of their sex. The animals were euthanized by exsanguination (regular slaughtering procedure) without anesthesia in the goat farm following a procedure approved by the Food Safety and Standards Authority of India. All the tissues were carefully dissected and embedded in optimal cutting temperature (OCT) media (Merck, India). The embedded tissues were snap‐frozen in liquid nitrogen and stored at −80°C. The muscle tissues were sectioned in cryotome (Invitrogen, USA) at 20 μm.

**TABLE 1:** Details of animal age grouping.

Sl. no.	Species and strain	Age group	Age
1	*Capra hircus* Black Bengal goat	Prenatal	Mid‐gestation
			Late‐gestation
2		Postnatal	1 week
			2 weeks
			1 month
			Adult (8–12 months)

### 2.2 H&E staining

The histoarchitecture of goat SkM sections was studied by staining with hematoxylin (SRL, India) and eosin (Himedia, India) (H&E). Thawed tissue sections were stained with hematoxylin for 1 min followed by tap water and distilled water washing. The sections were then stained with eosin for 1 min and washed with distilled water. The stained tissue sections were dehydrated using an upgraded series of alcohol, and the coverslip was mounted using DPX mounting media (SRL, India).

### 2.3 Succinate dehydrogenase staining

Succinate dehydrogenase (SDH) histochemical staining was performed using previously published protocols (Beermann et al., 1978; Pani et al., 2023). Tissue sections were stained in SDH incubating solution containing 0.2 M phosphate‐EDTA buffer, 100 mM sodium succinate, and 1.2 mM nitro‐blue tetrazolium at 37°C for 1 h, and washed with distilled water and air‐dried. Coverslips were mounted using gelatin aqueous mounting media.

### 2.4 Alkaline phosphatase staining

Alkaline phosphatase (ALP) histochemical staining was performed to study vascularization following published protocols (Engel & Cunningham, 1970). Tissue sections were stained using ALP incubating solution containing 100 mM Tris‐maleate buffer, 100 mM sodium‐1‐naphthyl phosphate monosodium salt, and fast blue stain at 37°C. The stained tissue sections were fixed with 1% acetic acid and then air‐dried in the dark. Coverslips were mounted using gelatine aqueous mounting media.

### 2.5 Immunohistochemistry

Immunohistochemistry (IHC) of MFN‐1 (Abcam, India, cat#ab104274), MFN‐2 (Merck, India, cat#SAB2108010), MFF (Cell Signalling Technology, U.S.A., cat# E5W4M), DRP‐1 (Cell Signalling Technology, U.S.A., cat#D8H5), SDH (Cell Signalling Technology, U.S.A., cat#5839), and OPA‐1 (Invitrogen, India, cat#PA1‐16991) was performed using commercially available kit (Pathnsitu biotechnologies, India., cat# SK‐4100) as per the protocol provided by the manufacturer. Briefly, the thawed tissue sections were subjected to peroxide blocking to stop in situ peroxidase activity. The antigen retrieval step was carried out by boiling the slides at 100°C in EDTA buffer (pH 9.0) solution for 20 min. The cooled slides were washed and blocked with a protein block provided by the manufacturer. Tissue sections were incubated with respective primary antibodies at 4°C overnight. The slides were then incubated with polyvalent secondary antibody provided in the kit for 30 min. Slides were washed and developed by DAB‐peroxide solution, provided in the kit. The tissue sections were counterstained with hematoxylin and then subjected to dehydration using an upgraded series of alcohol. The coverslip was mounted using DPX mounting media.

### 2.6 Proximity ligation assay

Proximity ligation assay (PLA) (Duolink®; DUO92101, Merck, India) detects two interacting proteins located within 40 nm by immunofluorescence detection. The assay was performed as per the instructions of the manufacturer. Briefly, the gastrocnemius tissue sections were incubated primary antibodies (MFN‐1: Abcam, India, cat#ab104274; MFN‐2: Merck, India, cat#SAB2108010) generated in two different species. This step is followed by incubation with secondary antibodies provided in the kit (known as plus and minus probes in kit) containing unique DNA strand which upon binding of antibodies to interacting proteins, form circular DNA by ligase treatment. This circular DNA is then amplified by rolling circle replication, and the interaction is visualized by fluorescently labeled complementary nucleotide probes. The tissues were then counterstained with DAPI.

### 2.7 Imaging and quantification

All the images were obtained under light microscopes: bright field (Leica, DM2000) and fluorescence (Invitrogen EVOS FL Digital Inverted Fluorescence Microscope) at 100× and 200× magnification. All the quantifications were performed using the Fiji (Image J 2) Software, following previously described methods (Crowe & Yue, 2019). The quantified values were represented in arbitrary unit (a.u.). In total, 45 images were selected for quantification in following manner: three animals/age group × 3 slides from each animal × 5 images from each slide with more or less similar number of fibers. For quantification of the ALP activity, the colored images were processed by the path “Image › Color › split channels.” After the RGB split, the red channel image was selected for further analysis as they represented black‐colored regions well (signifying ALP activity) and processed by the path Image > adjust > threshold (adjust according to the black region of the original image) > apply. The ALP activity was measured by selecting the mean gray value. SDH histochemical images were quantified by first converting the image into grayscale, following path image > type >8bit, and then measuring the mean gray value. The number of SDH^low^ fibers was counted manually by taking into fibers with the least expression of SDH. The fibers with intermediate expression were avoided while counting. The immunohistochemical images were quantified using a plugin named “Color deconvolution” which separates the colors of histologically stained images based on RGB splitting. The following path separated the channels: Image › Color › Color deconvolution > vectors (HDAB). The split images representing DAB coloration were further processed by path image > adjust > threshold (adjusted according to the original image) > apply. The protein expression was quantified by measuring the mean gray value.

### 2.8 Statistical analysis

All the statistical analyses were performed using Prism v5.0, and the quantified values were plotted as mean ± SD. The statistically significant differences were obtained between time points by one‐way ANOVA and between tissues by two‐way ANOVA.

## 3 RESULTS

### 3.1 Divergent blood‐vessel distribution pattern between locomotory and postural muscle groups

**FIGURE 1:**
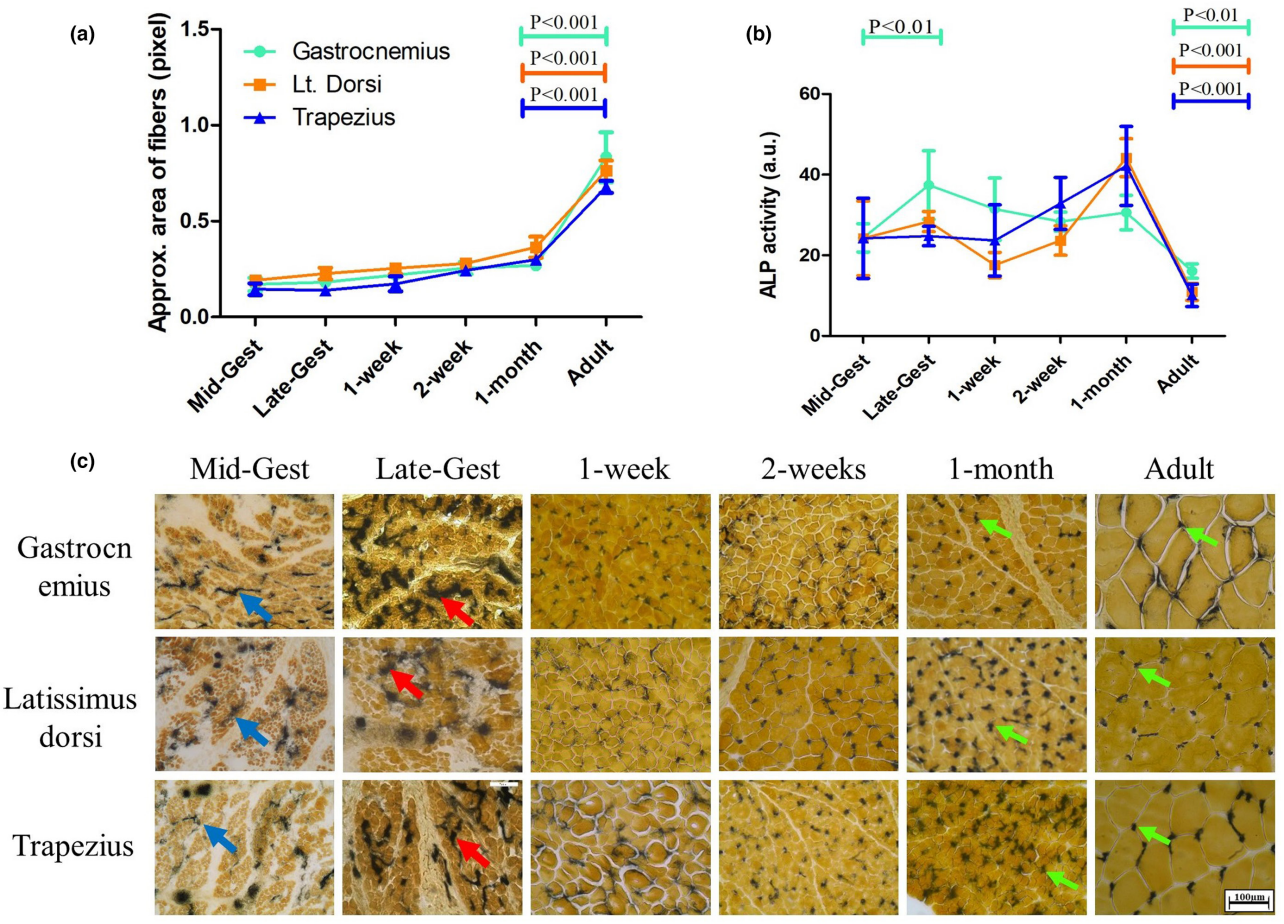
Fiber cross‐sectional area and vascularization during perinatal to adulthood transition: (a) The area of myofibers increase to a little extent till 1‐month postnatal age after which the fiber size increase in several folds (b) Quantified alkaline phosphatase (ALP) activity across age groups shows locomotory muscles (gastrocnemius) differ from postural muscles (trapezius and latissimus dorsi) in terms of vasculature density during development. During late gestation, gastrocnemius exhibit higher ALP activity than other two muscles. From 1 month, neonate to adult the ALP activity was significantly downregulated in all the muscles. (c) Larger blood vessels start to appear in mid‐gestation period (marked with blue arrow), become more branched during late gestation (pointed with red arrow) and precise fiber‐specific capillary network is observed after 1 month of postnatal age (demarcated with green arrow). In prenatal groups, all three muscle groups showed ALP‐positive staining for bigger blood vessels mostly in the epimysium and perimysium region. During the postnatal period, capillaries were enriched beside every myofiber with increasing age and attained maximum at 1‐month postnatal age. Significant differences between time points are represented in their respective color.

Histo‐anatomical observations are not well documented for goat SkM during perinatal development. In our previous study, we have observed the difference between neonate and adult muscle in terms of histoarchitecture, vascularization, and SDH activity (Senapati et al., 2023). However, to deduce the development and differentiation of SkM by considering these aspects, a more detailed developmental window is required. Therefore, we compared the histoarchitecture of stained SkM sections of gastrocnemius, latissimus dorsi, and trapezius muscle across the developmental groups. The H&E staining of all three muscle groups showed a relatively similar pattern of development (Figure S1). In all the prenatal groups, the number of nuclei was much higher with respect to the number of fibers. Interestingly, the mid‐ and late‐gestational muscles exhibited scattered fibers interspersed by tissue structures that do not gain staining and may represent non‐muscle cells. But, this interfibrillar space (or non‐muscle portion) reduces gradually in neonatal muscles from 1 week to 2 weeks and in a section, fibers become closely packed together (Figure S1). The growth in cross‐sectional area (CSA) of individual fiber till 1 month of age is minimal after which it undergoes rapid growth to reach maximum by adulthood (Figure 1a).

Next, we studied the development of vasculature inside SkM using ALP activity staining (Figure 1). During mid‐gestation, ALP activity in all three muscle groups showed irregular distribution and intensity. Also, the placement of ALP activity did not show a clear correlation with the position of the interspersed fibers. Whereas during late‐gestation a clear distinction was observed between latissimus dorsi and trapezius compared to gastrocnemius, which showed the highest ALP activity. In a sharp contrast, after birth the ALP activity gets organized and takes up a regular distribution pattern mostly localized to the convergence points of fibers. The distribution of ALP activity elsewhere is gradually eliminated during postnatal development and in adults is highly restricted to the convergence points.

### 3.2 Fibers having negligible SDH activity appear in locomotory muscles only after 1 month of birth

**FIGURE 2:**
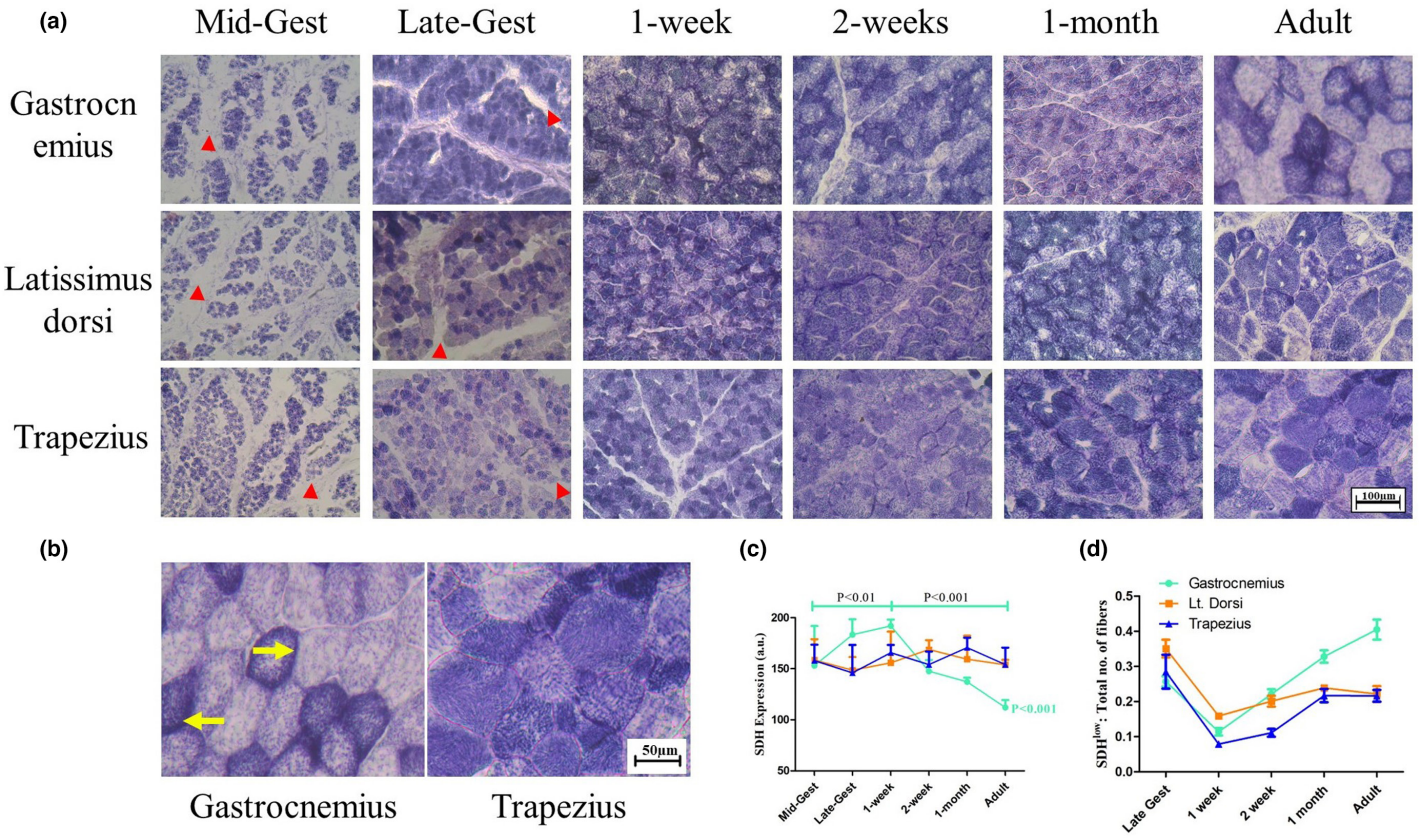
Distribution of mitochondrial activity in developing skeletal muscle: (a) SDH histochemical staining shows the oxidative difference between SDH^low^ and SDH^high^ fibers is more prominent during adulthood than in neonatal groups. In prenatal groups, the fibers are interspersed with non‐muscle portions not reacting to SDH staining and are visible as white areas demarcated with red triangles which was also observed in H&E staining (Figure S1). In all the postnatal groups, muscle fibers are closely packed without intervening white (non‐reactive) spaces. (b) Gastrocnemius showed more prominent distinction between SDH^low^ and SDH^high^ fibers while trapezius muscles showed a broad range of mitochondrial activity levels. SDH^high^ fibers in gastrocnemius show peripheral distribution of mitochondrial activity indicated by yellow arrow, whereas SDH^high^ fibers of trapezius exhibit uniform distribution of mitochondrial activity throughout the fiber. (c) The SDH activity was significantly higher in gastrocnemius compared to other two muscles during late gestation. Intriguingly, SDH activity in gastrocnemius drops drastically during later neonatal stages and adulthood. No significant variation was observed in SDH activity levels during pre‐ and postnatal periods in both latissimus dorsi and trapezius. (d) Among neonatal groups, the number of SDH^low^ fibers was found to be marginally altered whereas it was elevated in several folds in adult gastrocnemius. Significant differences between time points are represented in their respective color.

After finding unique vascularization patterns between the muscle groups, we analyzed the oxidative remodeling during the development of SkM by SDH histochemical staining used as an index of mitochondrial activity. During prenatal stages, the muscle fibers exhibit significant SDH activity in all three muscles and probably SDH^low^ fibers are extremely rare. In concurrence with the H&E data, the interspersed regions found in prenatal tissues did not react to SDH staining indicating those portions are non‐muscle structures. The gastrocnemius muscle shows higher SDH activity (labeled SDH^high^) during perinatal (late‐gestation and 1 week) stages than latissimus dorsi and trapezius. All the muscles showed maximum SDH staining during 1‐week postnatal period which declined rapidly in gastrocnemius. In contrast, latissimus dorsi and trapezius showed a comparatively flatter curve of declination of SDH staining from 1 week to adulthood. The fibers with a low SDH activity (labeled as SDH^low^) got enriched in adult gastrocnemius that makes the muscle to bear both SDH^low^ and SDH^high^ fibers. In contrast, abundance of SDH^low^ fibers was found to be much lower in both the adult postural muscles, where SDH activity of individual fibers exhibits a broad range from low to high (Figure 2). Another interesting observation was found in mitochondrial activity distribution pattern at individual fiber level of adult locomotory versus postural muscles. While gastrocnemius exhibited high abundance of peripheral SDH activity in SDH^high^ fibers, trapezius showed uniform distribution of SDH activity throughout SDH^high^ fibers. Further, in gastrocnemius mitochondrial activity remained uniformly distributed during the pre‐and‐postnatal stages up to 1 month. These observed alterations in SDH histochemical staining were also supported by SDH immunohistochemical staining (Figure S2).

### 3.3 Mitochondrial fission proteins exhibit homogenous expression in neonates whereas restricted to the periphery in adult myofiber

**FIGURE 3:**
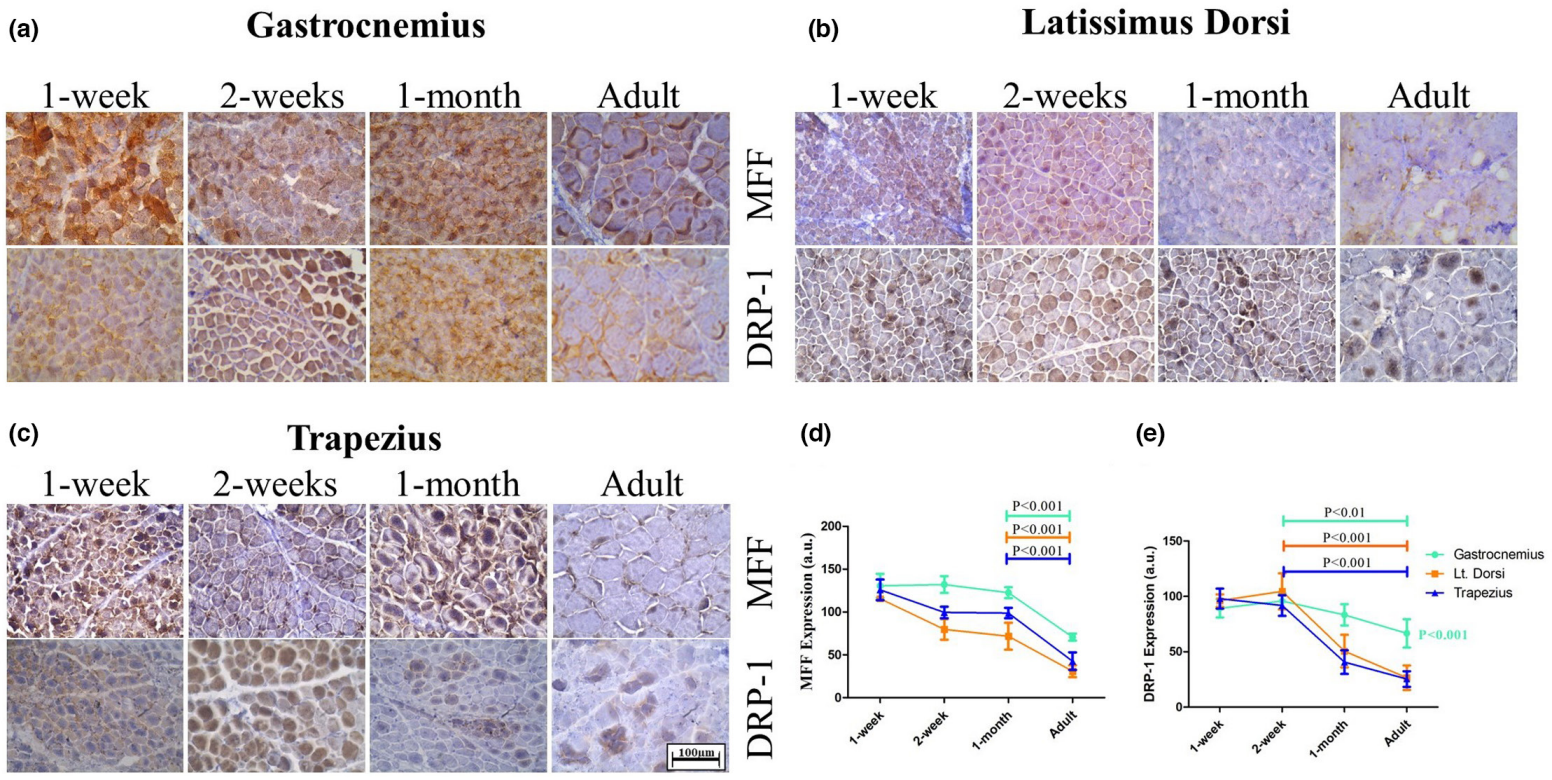
Expression of mitochondrial fission‐associated proteins during postnatal development of skeletal muscle: Expression of MFF and DRP‐1 during postnatal developmental window of goat in (a) gastrocnemius, (b) latissimus dorsi, and (c) trapezius. Both proteins were expressed all over the myofiber during the early postnatal period while they are predominantly expressed in the subsarcolemmal region during adulthood. Quantified expression of (d) MFF and (e) DRP‐1 shows higher expression of both proteins in adult gastrocnemius muscle. Significant differences between time points are represented in their respective color.

Difference in mitochondrial SDH activity during development among the three muscle groups prompted us to study mitochondrial dynamics during these developmental stages. Mitochondrial fission and fusion are the two crucial processes regulating its dynamics and metabolic properties. Also, recent studies have shown the proteins associated with fission and fusion to be crucial regulators during SkM development and differentiation. Any alteration in their expression leads to change in SDH^low^–to‐SDH^high^ fiber ratio (Wang et al., 2022; Yasuda et al., 2023). Therefore, to check the rate of mitochondrial fission across different ages we choose two crucial proteins involved in this process: MFF and DRP‐1 (Figure 3). Interestingly, the proteins showed a high level of expression during the perinatal period as massive mitochondrial remodeling was observed during this period. In early postnatal groups (1 week and 2 weeks), both the fission‐associated proteins were at the peak after which it was significantly downregulated by 1 month in all the three muscle groups. Expression of MFF in gastrocnemius was found to be higher than the other two muscles during all stages. The gastrocnemius muscle also exhibited higher expression of DRP‐1 in 1 month old and adults compared to the other two muscles. All three muscles showed higher DRP‐1 expression during neonatal stages and underwent a progressive downregulation. Another important observation made here is that both MFF and DRP‐1 were expressed evenly in neonatal (up to 1 month) myofibers whereas expression became predominantly localized to the periphery (subsarcolemmal area) in adult myofibers (Figure 3).

### 3.4 Mitochondrial fusion proteins are evenly distributed in a fiber‐specific manner while MFN‐1‐MFN‐2 interaction exhibits peripheral localization

**FIGURE 4:**
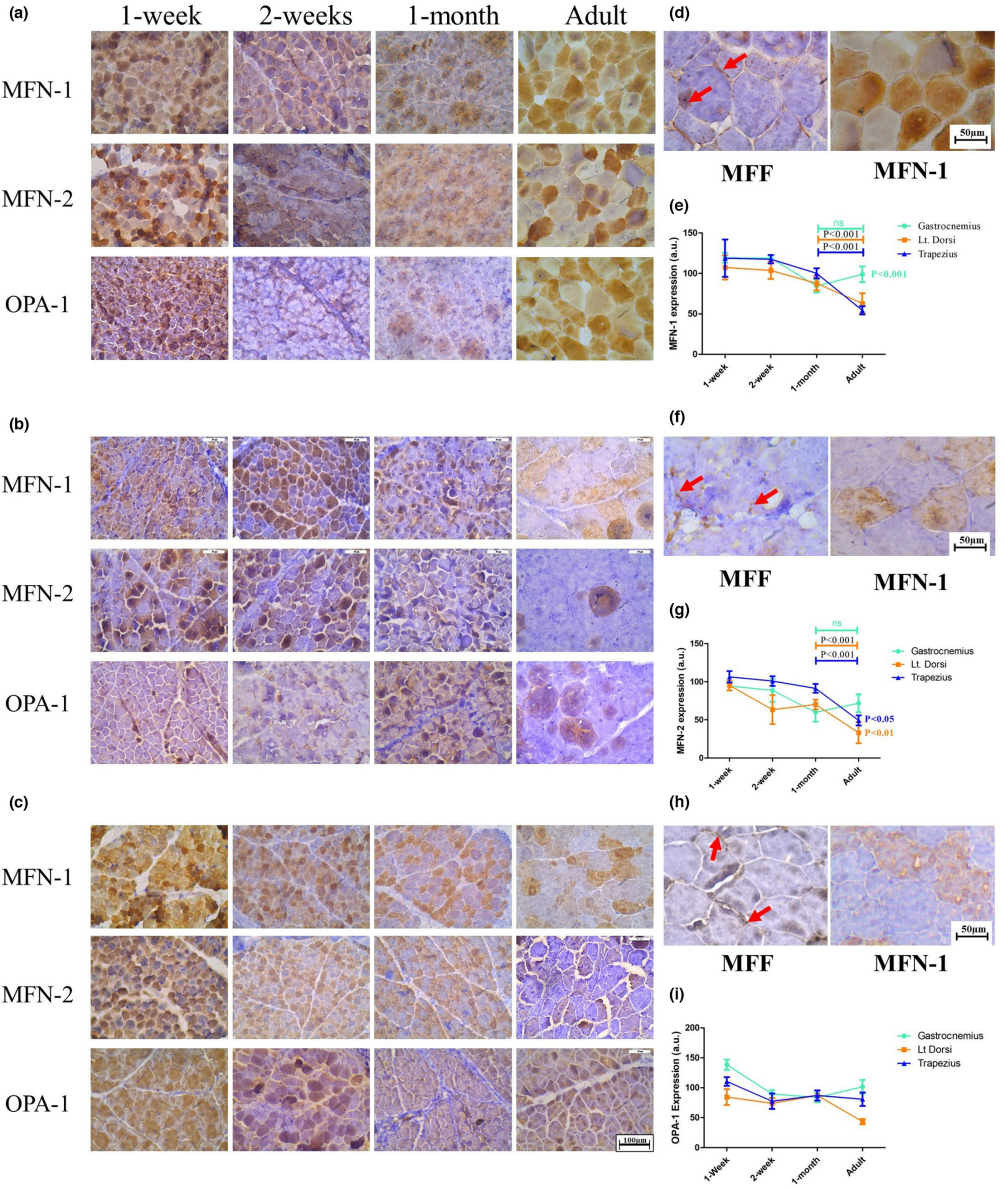
Alteration in distribution of mitochondrial fusion proteins during postnatal development of skeletal muscle: Expression of MFN‐1, MFN‐2, and OPA‐1 during postnatal development of goat in (a) gastrocnemius, (b) latissimus dorsi, and (c) trapezius. (d, f, h) The expression of all three proteins was centralized to a few specific fibers in all three muscle groups during adulthood, in contrast to MFF which is mostly limited to peripheral regions of myofibers. Quantified expression shows outer mitochondrial membrane proteins: (e) MFN‐1 and (g) MFN‐2 follow similar trendlines during the developmental window. (i) OPA‐1 follows a different pattern which is inner mitochondrial membrane protein.

**FIGURE 5:**
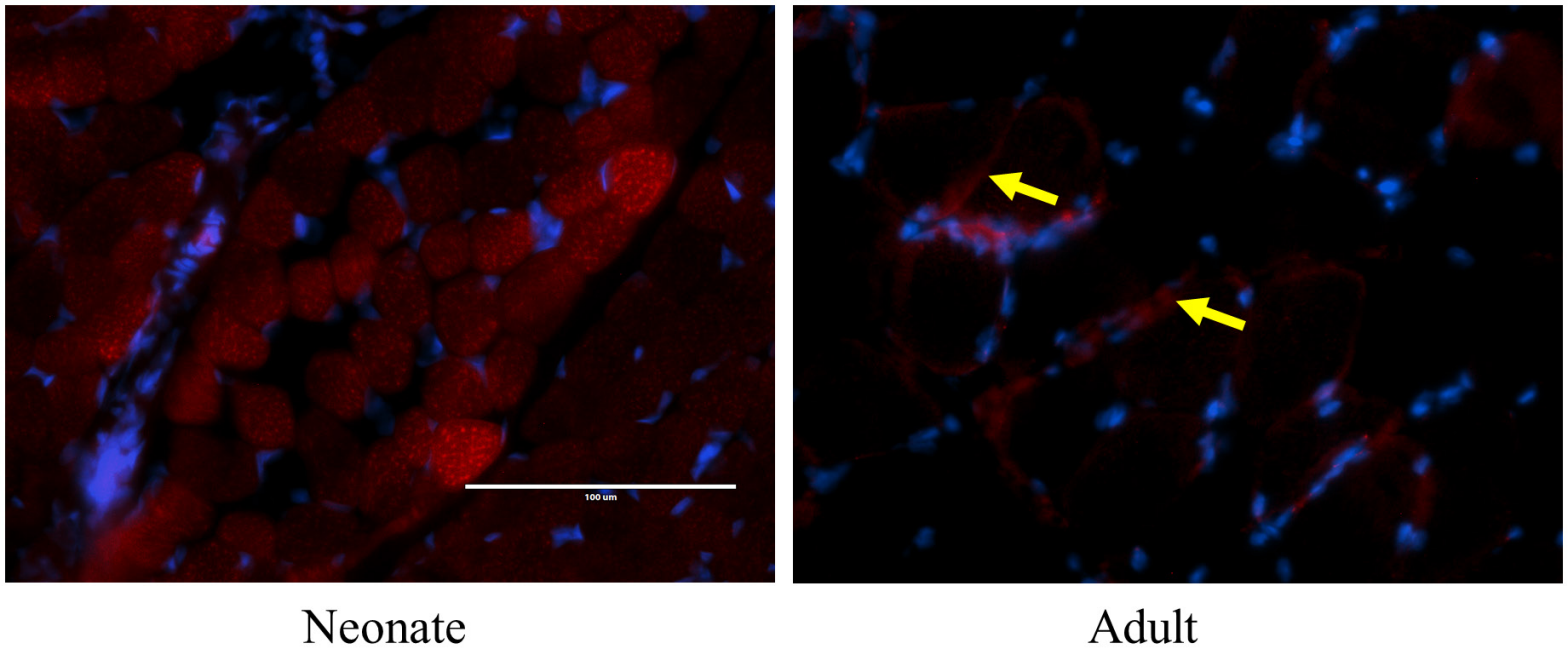
Localization of protein–protein interaction between MFN‐1 and MFN‐2 in the neonate and adult skeletal muscle: Proximity ligation assay (PLA) of MFN‐1 and MFN‐2 shows they are expressed in small dots spread throughout the fiber during the 1‐week postnatal period while adult myofibers show more elongated expression in the subsarcolemmal region.

Similarly, to study the mitochondrial fusion process we have checked the immunohistochemical expression of crucial mitofusin proteins like MFN‐1, MFN‐2, and OPA‐1. The expression pattern of MFN‐1 and MFN‐2 was quite similar across different age groups for all three muscles. The expression of both proteins seemed to be restricted to some specific fibers. While some of the fibers expressed these proteins at a high level, some others expressed at intermediate levels and the rest showed a very minor expression in the same muscle (Figure 4a–c). During adulthood, the fibers expressing either of the proteins showed a uniform distribution throughout in contrast to the expression of MFF which was primarily localized in the peripheral regions of all the three muscles (Figure 4d,f,h). The expression of MFN‐1 and MFN‐2 was high in muscles from 1‐week and 2‐week aged animals after which the expression was downregulated (Figure 4e,g). While postural muscles showed downregulated expression in adults gastrocnemius showed significantly higher expression. OPA‐1 also exhibited a similar expression profile being high during neonatal stages and gradual downregulation in postural muscles, while upregulation during adulthood (Figure 4i). Next, we were curious to co‐localize MFN‐1 and MFN‐2 in the developing muscles, for which we used PLA to analyze the interaction. Our result demonstrates that the MFN‐1‐MFN‐2 interaction is evenly distributed throughout the neonatal (1 week) myofibers. Intriguingly, this interaction becomes restricted to the peripheral zones in the muscle fibers of adult goats (Figure 5), which may indicate that MFN‐1‐MFN‐2 interaction is a critical factor in the developmental regulation of SkM.

## 4 DISCUSSION

Skeletal muscle increases its mass during development by both increase in the number (hyperplasia) (McCoard et al., 2001; Ren et al., 2011) and size (hypertrophy) of fibers (Goss, 1966). SkM located at different positions of the body are functionally divergent, hence undergo differential developmental programming. SkM myogenesis starts during early‐to‐mid gestation and undergoes rapid myonuclear division (Bentzinger et al., 2012). Our data corroborate, as the number of nuclei was several folds higher than the number of fibers only in the prenatal groups. The differentiated myocytes make up a smaller volume of the whole muscle during prenatal period as they are surrounded by undifferentiated progenitor cells, extracellular matrix (mostly collagen fibers), other interspersed non‐muscle cells (Leichsenring et al., 2021). This non‐muscle portion is significantly reduced during the early postnatal period in all mammals. However, this myocyte differentiation process varies across species in terms of developmental timeline (Bensamoun et al., 2006; Binzoni et al., 2001; Draeger et al., 1987; Punkt et al., 2004). The size of muscles increased rapidly during the late prenatal to early postnatal period without a significant increase in fiber CSA. Interestingly after 1 month, the CSA of muscle fibers increased significantly by several folds which coincided with the earlier studies from human samples that proposed fiber hypertrophy starts after 8 weeks (Barbet et al., 1991; Sewry et al., 2021). In a sharp contrast, rodent muscle fiber hypertrophy starts close to weaning age that indicates in case of goats it is probably achieved quite early on during postnatal development. (Bérard et al., 2011; Lefaucheur & Gerrard, 2000; Senapati et al., 2023). This suggests early postnatal development of SkM primarily relies on hyperplasia whereas at a later stage on hypertrophy (Bérard et al., 2011).

We observed that during the prenatal period, the SkM are mostly supplied with larger blood vessels and the majority of myofibers are devoid of capillaries. The formation of the capillary network to supply individual fibers occurs between 1‐week to 1‐month postnatal period. Major vasculogenesis was found to be higher during the late‐gestational period in primary locomotory muscles like gastrocnemius, which might be to assist the precocial character of goats; analogous to other precocial larger mammals like horse/sheep/pig/cow. In contrast, our previous study on rats showed locomotory muscles undergo major vasculogenesis during postnatal (~1 week after birth) periods (Senapati et al., 2023). The study from Stingl and Rhodin also shows the capillary network gets established after 3 weeks of postnatal development which is much later as compared to goats in terms of developmental/lifespan trajectory (Stingl & Rhodin, 1994). Another interesting point is that during the late postnatal (1 month after birth) period trapezius and latissimus dorsi exhibited higher vascularization supplying individual fibers, which might be to promote the oxidativeness of these postural muscles as observed for cattle species (Liu et al., 1995). With the increase in fiber CSA during adulthood, the vascularization per unit area in all the muscles is reduced dramatically.

Vascularization and oxidative metabolic capacity of SkM are interdependent on each other (Figure S3) (Bal et al., 2017; Iversen et al., 2011; Joanisse et al., 2017). Prenatal SDH activity was concomitant with the ALP activity, indicating that the elevated oxidative capacity of locomotory muscles during the late‐gestational period is accompanied by increased vascularization. The goat SkM maturation also indicated to be closer to humans than rodents based on SDH activity (Draeger et al., 1987; Leichsenring et al., 2021; Punkt et al., 2004). During early postnatal period, in most of the animals SkM fiber type is unspecified and all the myofibers contain evenly distributed SDH activity throughout individual fibers. With increasing age, myofibers display SDH activity depending on their fiber type and location in the body. During adulthood, locomotory and postural muscles show different patterns of muscle tone to support their respective function. The locomotory muscles contain either SDH^high^ or SDH^low^ myofibers during adulthood that provide opportunity for being recruited during different activities. The increased locomotory behavior with increasing age is definitely a crucial modulator for the enrichment of SDH^low^ (probably glycolytic) in gastrocnemius. In contrast, the postural muscles are rich in fibers with intermediate SDH activity which may provide energy for them to remain in an isometrically contracted state. The SDH^high^ fibers in adult gastrocnemius exhibit peripheral distribution of mitochondrial activity that may enable the fiber to localize contractile capacity to the central portion and might be beneficial for locomotion. In addition, the above contrasting features between neonate and adult fibers might be related to differential preference of substrates especially in the locomotory muscles.

Our data provide evidence that mitochondrial fission and fusion proteins exhibit unique developmental regulation in their expression and fiber‐specific distribution. They are expressed plentifully throughout the fiber indicating high mitochondrial fission‐fusion during late gestation and early postnatal stages (Kim et al., 2019; Vidyadharan et al., 2022). Moreover, their expression has been shown to play a crucial role in neonatal myogenesis in mice. Depletion of either fission or fusion process has been shown to alter the ratio between glycolytic and oxidative fibers and induce atrophy (Wang et al., 2022; Yasuda et al., 2023). MFN‐2 has been shown to play crucial role in muscle development in mice models. The peripheral localization of DRP‐1 and MFF during adulthood (especially in locomotory muscle) may indicate fission processes become restricted to the subsarcolemmal region. This may indicate subsarcolemmal mitochondria (SSM) undergo more rapid fission during adulthood, whereas interfibrillar mitochondria (IFM) are comparatively more stable (Koves et al., 2005). As IFM has already established mitochondrial reticulum and is an integral part of myofibril during adulthood, its modification will lead to structural instability (Manneschi & Federico, 1995; Willingham et al., 2021). All three mitochondrial fusion proteins, MFN‐1, MFN‐2, and OPA‐1, showed fiber‐specific evenly distributed expression patterns even in adult muscles, which is more prominent in gastrocnemius. This indicates that there is a constant requirement of mitochondrial fusion throughout the fiber CSA encompassing both IFM and SSM which is even more important in muscles with primary locomotory function. With the data from PLA, we for the first time demonstrate that during the early postnatal period, MFN‐1/MFN‐2 interaction happens throughout the CSA of neonatal myofibers. However, the MFN‐1/MFN‐2 interaction in adult fibers is limited to the subsarcolemmal area, where SSM is abundantly present. Recent studies show that MFN‐1 and MFN‐2 serve as tethers between mitochondria and SR establishing a sarco‐mitochondrial network (Boncompagni et al., 2020). Also, their interaction has been shown in trans‐mitochondrial fusions (Chen et al., 2003). So, it seems possible that SSM relies more on this tethering interaction than that of IFM. Further research will validate the inter‐organellar interaction and such a special arrangement of MFN‐1/MFN‐2 interaction might help in location‐specific energy distribution in an individual fiber.

## AUTHORS CONTRIBUTIONS

SP and NCB conceptualized the idea and conceived the study. SP, US, BP, BS, GS, and PP collected the animal tissues and performed the experiments. SP, US, and SR performed all the quantifications, and SP, US, and KGA performed the tissue sectioning. SP and NCB analyzed the data and wrote the manuscript. All authors read and reviewed the manuscript.

## FUNDING INFORMATION

This work has been supported by Science and Engineering Research Board (SERB), DST, India (grant no. ECR/2016/001247) and DBT, India (grant no. BT/RLF/Re‐entry/41/2014 and BT/PR28935/MED/30/2035/2018) to N.C.B. S.P. and G.S. are recipients of Senior Research Fellowships from Indian Council of Medical Research (ICMR) vide grant nos 45/3/2019/PHY/BMS and 45/9/2020‐PHY/BMS, respectively. B.S. is a recipient of JRF from the Council of Scientific and Industrial Research (CSIR), India with grant no 09/1035(0011)/2017‐EMR‐I. P.P. is a recipient of JRF from DST, Ministry of Science & Technology, INDIA with grant no DST/INSPIRE Fellowship/2018/IF180892.

## CONFLICT OF INTEREST STATEMENT

We declare we have no competing interests.

## ETHICS APPROVAL STATEMENT

All the animal experimentation procedures were performed in accordance with the guidelines of the institutional animal ethics committee (IAEC) of the School of Biotechnology, KIIT with approval number: KSBT/IAEC/2019/MEET‐1/A13.

## Supporting information


Figure S1.

Figure S2.

Figure S3.

## Data Availability

The data generated in the current study are not publicly available but are available from the corresponding author on reasonable request.
