# Supplementary figures and images for: Developmental dynamics of mitochondrial fission and fusion proteins in functionally divergent skeletal muscles of goat
Source: Physiol Rep. 2024 Jun 3;12(11):e16002. doi: 10.14814/phy2.16002 (PMC11148127; doi:10.14814/phy2.16002)

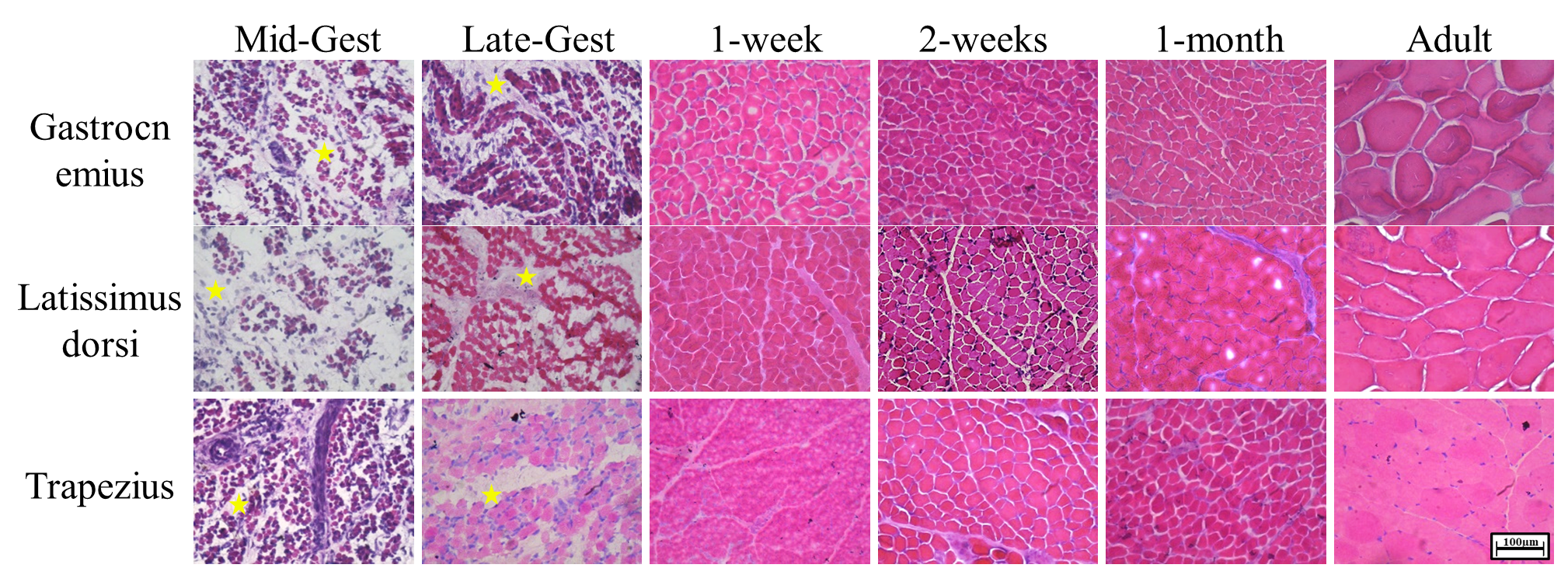

Supplement: Supplementary file 1 — Figure S1. [file PHY2-12-e16002-s001.tif]

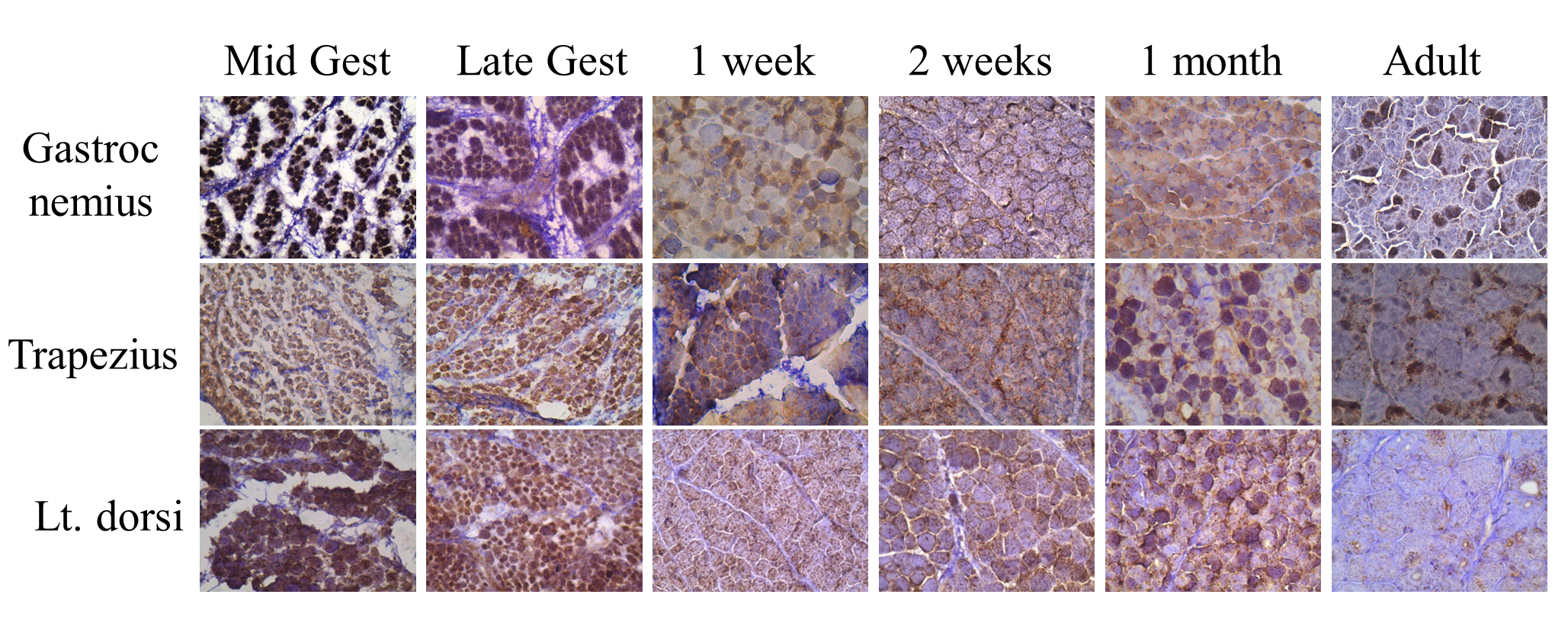

Supplement: Supplementary file 2 — Figure S2. [file PHY2-12-e16002-s002.tif]

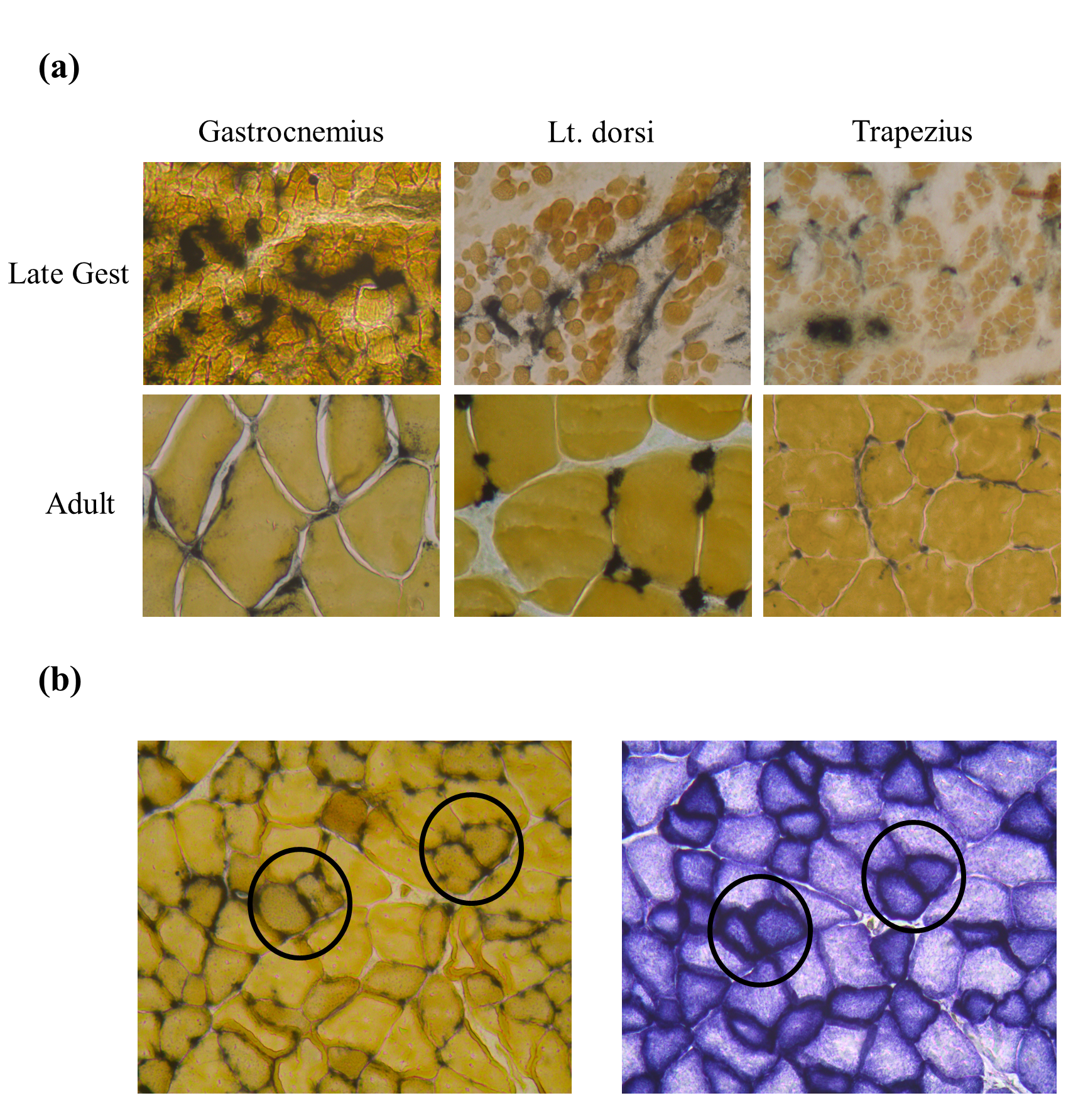

Supplement: Supplementary file 3 — Figure S3. [file PHY2-12-e16002-s003.tif]
